# Reported safety events from ultrasound enhancing agents: a critical reappraisal

**DOI:** 10.1186/s44156-026-00127-1

**Published:** 2026-07-13

**Authors:** Jordan B. Strom, Nicholas Spetko, Constance E. M. Angell-James, Madeline A. Cassidy, Jessica L. Stout, Morgan L. Winburn, Rebecca Lee, Alecia Bradley, Kevin Moreau, Venkateshwar Polsani, Cosby A. Stone

**Affiliations:** 1 Division of Cardiology, Department of Medicine, Beth Israel Deaconess Medical Center, 375 Longwood Avenue, 4th Floor, Boston, MA 02215 USA; 2 Harvard Medical School, Boston, MA USA; 3 Richard A. and Susan F. Smith Center for Outcomes Research in Cardiology, Boston, MA USA; 4 Division of Infectious Diseases, Department of Medicine, Vanderbilt University Medical Center, Nashville, TN USA; 5 Piedmont Hospital System, Atlanta, GA USA; 6 Division of Allergy, Pulmonary, and Critical Care Medicine, Department of Medicine, Vanderbilt University Medical Center, Nashville, TN USA

**Keywords:** Ultrasound enhancing agents, Safety

## Abstract

**Background:**

Reported adverse events (AEs) to the Food and Drug Administration Adverse Event Reporting System (FAERS) have suggested an increased rate of serious AEs (SAEs) during the COVID-19 pandemic, but the extent to which this may be related to overall changes in reporting during this time period is uncertain. Accordingly, we aimed to evaluate trends in SAE reporting across commercially available ultrasound enhancing agent (UEA) brands as a function of overall trends in AE reporting.

**Methods:**

We retrospectively analyzed the FAERS public database, 2014–2024, to evaluate risks of UEAs overall and by brand, compared to similar contrast media.

**Results:**

Between 2014 and 2024, 21,960,760 AEs were reported to FAERS, of which 11,450,891 (52.1%) were categorized as SAEs. Overall SAE reports to FAERS increased from 678,953 in 2014 to 1,368,393 in 2021 before subsequently declining to 1,065,845 in 2024 (-7.9% change from 2021 to 2024). During the same period, overall death reports to FAERS increased from 124,055 in 2014 to 195,207 in 2018 before declining to 147,046 in 2024. During this period of decline, there was a 23.9% relative increase in SAEs to UEAs which peaked in 2023 at 350 before declining to 326 in 2024. Deaths attributed to SAEs increased from 1 in 2014 to 19 in 2023 before declining to 9 in 2024. Overall, these data suggest that 11.2% of the observed increase in SAEs to UEAs can be attributed to reporting changes. Despite changes in relative risks for SAEs, absolute SAE rates remained small and lower than other types of contrast media.

**Conclusions:**

In this analysis of the FAERS dataset, 2014–2024, 11.2% of SAEs to UEAs were attributable to temporal changes in AE reporting. Absolute risks are small and declining, suggesting broad safety of UEAs as a class. Collectively, these results support continued use of UEAs, but motivate improved safety screening and preparedness to mitigate small but existing risks.

**Supplementary Information:**

The online version contains supplementary material available at 10.1186/s44156-026-00127-1.

## Introduction

Ultrasound enhancing agents (UEAs), consisting of gas-filled phospholipid-shelled microspheres whose resonance and backscatter properties provide effective myocardial tissue contrast, have become an indispensable part of the ultrasound armamentarium [1, 2]. UEAs have a multitude of clinical uses in cardiology (e.g. improving endocardial border resolution, assessing myocardial and mass perfusion, and evaluating apical pathology on echocardiograms [1]) and radiology (e.g. identification and characterization of masses, monitoring of inflammatory and neoplastic gastrointestinal or renal diseases [3, 4]) with novel applications actively being explored [1].

While historically, UEAs have been considered amongst the safest of all contrast media [5], with a fixed but small rate of serious adverse events (SAEs) of approximately 1:10,000–1:15,000 attributed to idiosyncratic complement activation related pseudoallergy (CARPA) reactions [5–8], recent reports from individual centers [9–11] have questioned whether both the class of agents as well as specific brands may be of elevated risk in the contemporary era. A prior published analysis [12] of the US Food and Drug Administration (FDA)’s Adverse Event Reporting System (FAERS), a large and public database of reported adverse events to all therapeutics, 2019–2023, has suggested there may be differences in safety across brands, though this analysis was subject to a number of methodologic limitations [13], furthering the uncertainty on this topic. At the same time, large unselected claims-based analyses have suggested that SAEs to UEAs have remained unchanged during the same time period and were similar cross brands [14], leading to confusion in how to reconcile these discordant findings.

As the number and brands associated with reported UEA-related SAEs to the FDA could reflect changes in overall reporting over the period of time of the COVID-19 pandemic and growth in UEA utilization rather than a worsening safety profile, we aimed to re-analyze the FAERS dataset to evaluate this hypothesis further. We speculated that changes in the background rate of adverse event (AE) reporting in FAERS could potentially explain, at least partially, prior discrepant study findings and help contextualize the contemporary safety of these clinically important agents as a class.

## Methods

### Study design and population

We performed a retrospective analysis of publicly available data inclusive of all AEs reported to FAERS, 2014–2024. The FAERS dataset [15] contains AE reports submitted by pharmaceutical companies, healthcare providers, and consumers as well as medication error reports and product quality complaints resulting in AEs and is designed to support post-marketing safety surveillance efforts. The FAERS database spans all FDA-regulated drugs and biologic medications (including non-imaging-related agents), but not devices [15]. We chose to evaluate reports from 2014 to 2024 as this represents the period of time over which an increased number of SAEs to UEAs were reported [11]. Data from 2025 were not included as they were incomplete at the time of analysis. No other exclusions were applied.

### Ethics, consent to participate, and consent to publish

Given only publicly-available, retrospective, and deidentified data were evaluated, the analysis was considered exempt from Institutional Review Board review.

### Definition of exposure

The FAERS dataset was queried for AEs attributed to any of the 3 comercially-available UEA brands in the US, including Optison™ (GE Healthcare, Waukesha, WI), Definity™/Luminity™ (Lantheus Medical Imaging, Billerica, MA), and Lumason™/Sonovue™ (Bracco Imaging S.p.A., Milan, Italy). Given the US-centric nature of the analysis, US brand names are used throughout the manuscript. Information on Lumason™ was only available after FDA regulatory approval in 2016. As there was only one report of an SAE attributed to multiple agents, this report was excluded from analyses. As a negative control group, we evaluated AEs attributed to non-PEGylated contrast agents including gadolinium contrast (gadobutrol [Gadavist™, Bayer AG, Leverkusen, Germany], gadoteridol [ProHance™, Bracco S.p.A., Milan, Italy], gadoterate meglumine [Clariscan™, GE Healthcare, Waukesha, WI], gadoterate meglumine [Dotarem™, Guerbet, Villepinte, France]) and iodinated contrast (including iohexol [Omnipaque™, GE Healthcare, Waukesha, WI], iodixanol [Visipaque™, GE Healthcare, Waukesha, WI], iopamidol [Isovue™, Bracco S.p.A., Milan, Italy], iopromide [Ultravist™, Bayer AG, Leverkusen, Germany], and ioversol [Optiray™, Guerbet, Villepinte, France]). As a positive control group, we evaluated AEs to Neulasta™ (Amgen, Thousand Oaks, CA), a recombinant human filtrastim protein conjugated to PEG .

### Definition of outcome

The FAERS dataset qualifies all reactions as either serious, non-serious, or fatal. Serious AEs include those resulting in death, hospitalization, disability or permanent impairment, birth defects, or required intervention to prevent permanent impairment [15]. Serious AEs also include those considered life-threatening or otherwise could jeopardize the welfare of the patient and include anaphylactic/anaphylactoid reactions [15]. While this definition of SAEs includes fatal events, death events are additionally categorized separately.

### Covariates

Limited covariate information is present in the FAERS database but includes the date of the event, the suspected agent responsible for the AE, the patient’s age, sex, and weight [15]. Of note, the unit of analysis in the dataset is the number of reactions, not the number of individuals and as such it is possible for a given individual to be included more than once. As it is not possible to distinguish unique individuals in the dataset, each reaction was assumed to come from a unique person for the purposes of analysis [15]. Furthermore, each reaction reported was assumed to occur only once in the FAERS dataset [15].

### Statistical analysis

Limited baseline characteristics are presented using counts and percentages for categorical variables and means and standard deviations for continuous variables and compared across UEA brands using Chi squared and one-way analysis of variance respectively. Stacked bar graphs were used to display the distribution of overall reactions in the FAERS dataset in aggregate and stratified by year and reaction severity. Similar methods were used to display the distribution of reactions to UEAs in aggregate and stratified by year, brand, and reaction severity. Line plots were used to plot the rate of SAEs to UEAs per 10,000 reported events, separately considering all AE reports vs. all SAEs as the denominator. Poisson trend tests were used to estimate if there was a significant monotonic decrease or increase in SAE rates over the study period. Univariable Poisson regression was used to model the rate ratio (RR), 95% confidence interval (CI), and Wald p-value for SAEs to UEAs as a function of overall AEs vs. only SAEs using 2014 as the reference year. Lastly, the number of SAEs as a function of total UEA reactions was estimated by year and brand. Equivalent analyses were performed stratified by UEA brand and among gadolinium and iodinated contrast as negative controls and pegfilgrastim (i.e. Neulasta™) as a positive control. All analyses were conducted using JMP Pro v18 (SAS Institute, Cary, NC) using a two-tailed p-value < 0.05 to define significance.

### External comparison cohort

Overall trends were confirmed in complete electronic health record data from across multiple centers (37 echo labs including ambulatory and inpatient facilities) in the Piedmont Health System in Atlanta, GA, 2021–2024. This integrated health network was selected due to their catchment area across the entire state of Georgia, widespread use of UEAs, and detailed tracking of AEs. As per routine practice, all AEs reported across Piedmont are entered into a database management system and serious AEs are independently adjudicated by a single cardiac sonographer with prior training in AE recognition and classification (K.M.). Rates of serious and non-serious AEs to UEAs per 10,000 administrations are presented using stacked bar graphs and in tabular format.

## Results

### Overall reactions in FAERS

**Fig. 1:**
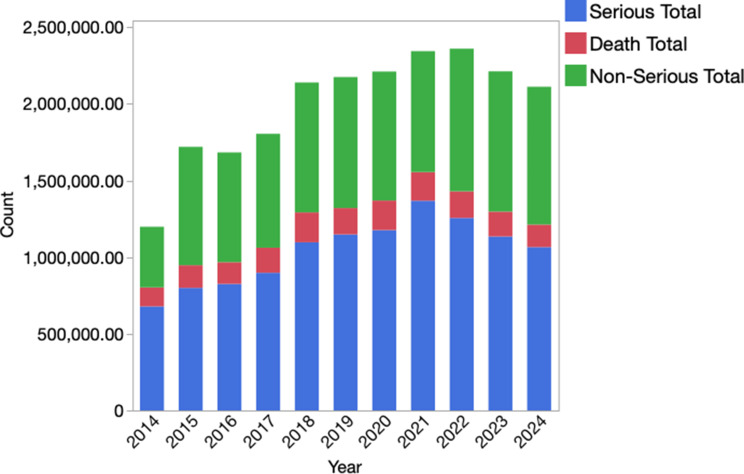
Overall numbers of reported adverse events to FAERS, 2014–2024. Legend: Shown is a stacked bar graph demonstrating the overall number of reported adverse events to the U.S. Food and Drug Administration Adverse Event Reporting System (FAERS) from 2014–2024. Blue bars indicate events classified as serious by FAERS, red bars indicate events resulting in death, and green bars indicate events classified as non-serious

Between 2014 and 2024, a total of 21,960,760 AEs were reported to the FAERS database including 11,450,891 SAEs (52.1%), 8,702,929 non-serious reactions (39.6%), and 1,806,940 deaths (8.2%). The number of reported AEs of all severities increased from 2014 to 2022 before falling from 2022 to 2024, similar to the number of SAEs and non-serious AEs, while the number of deaths peaked in 2018 (Table 1; Fig. 1).

**Table 1:** Overall adverse event reports to FAERS by year and severity

Year	Total Reports (*N* = 21,960,760)	Serious Reports (*N* = 11,450,891)	Non-Serious Reports (*N* = 8,702,929)	Deaths (*N* = 1,806,940)
2014	1,198,421	678,953	395,413	124,055
2015	1,719,763	799,861	772,024	147,878
2016	1,683,307	825,676	715,810	141,821
2017	1,804,751	898,413	743,206	163,132
2018	2,139,736	1,096,978	847,551	195,207
2019	2,175,069	1,148,573	854,428	172,068
2020	2,211,092	1,176,882	841,482	192,728
2021	2,344,116	1,368,393	788,871	186,852
2022	2,360,165	1,256,125	930,268	173,772
2023	2,212,442	1,135,192	914,869	162,381
2024	2,111,898	1,065,845	899,007	147,046

Legend: Shown are the total number of adverse event reports, serious adverse reports, non-serious adverse event reports, and adverse events resulting in death reported to the U.S. Food and Drug Administration Adverse Event Reporting System (FAERS) from 2014–2024 by year. N = number of reports

### Reactions to UEAs

**Fig. 2:**
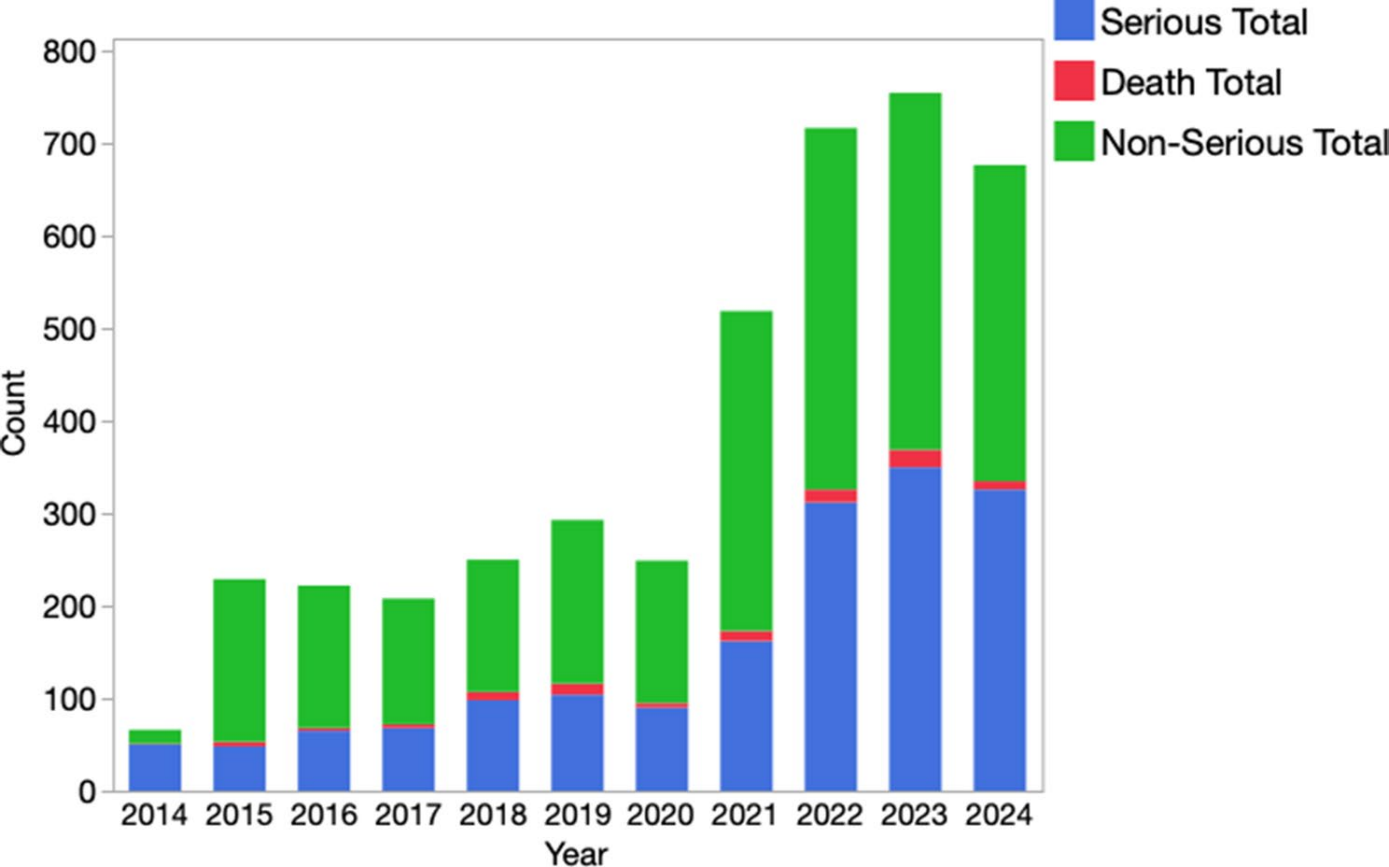
Overall numbers of reported adverse events to ultrasound enhancing agents in FAERS, 2014–2024. Legend: Shown is a stacked bar graph demonstrating the number of adverse events to ultrasound enhancing agents (UEAs) reported to the U.S. Food and Drug Administration Adverse Event Reporting System (FAERS) from 2014–2024. Blue bars indicate events classified as serious by FAERS, red bars indicate events resulting in death, and green bars indicate events classified as non-serious

During the same time period, 4,185 AEs were reported that were attributed to UEAs, including 1,673 SAEs (40.0%), 2,420 non-serious AEs (57.8%), and 92 deaths (2.2%). While non-serious AEs peaked in 2022, similar to overall trends, SAEs and deaths peaked in 2023 before declining (Fig. 2; Table 2). Of this total, 3,076 AEs (73.5%) were from Definity™, 1,042 (24.9%) from Lumason™, 66 (1.6%) from Optison™, and one attributed to more than one agent. Those with reported reactions to Definity™ were older (60.6 ± 16.5 years) than Lumason™ (58.2 ± 16.5 years) or Optison (58.2 ± 13.8 years) (*p* = 0.004) and significantly more likely to be female (Definity™, 54.3%; Lumason™, 36.0%; Optison™, 43.9%; *p* < 0.001) but had no differences in weight (*p* = 0.22) (Table 3) For reported SAEs, similar sex patterns were observed but there was no difference across brands in age (*p* = 0.24) or weight (*p* = 0.51). No significant differences in death events were noted across sex (*p* = 0.20), age (*p* = 0.42), or weight (*p* = 0.34) categories.

**Table 2:** Overall adverse event reports to FAERS attributed to ultrasound enhancing agents by year and severity

Year	Total Reports (*N* = 4093)	Serious Reports (*N* = 1673)	Non-Serious Reports (*N* = 2420)	Deaths (*N* = 92)
2014	65	50	15	1
2015	224	48	176	5
2016	219	65	154	3
2017	204	68	136	4
2018	241	98	143	9
2019	281	104	177	12
2020	244	90	154	5
2021	508	162	346	11
2022	703	312	391	14
2023	736	350	386	19
2024	668	326	342	9

Legend: Shown are the total number of adverse event reports, serious adverse reports, non-serious adverse event reports, and adverse events resulting in death reported to the U.S. Food and Drug Administration Adverse Event Reporting System (FAERS) and attributed to ultrasound enhancing agents (UEAs) from 2014–2024 by year. N = number of reports

**Table 3:** Characteristics of individuals experiencing reactions in FAERS by brand of ultrasound enhancing agent

	Definity (*N* = 3076)	Lumason (*N* = 1042)	Optison (*N* = 66)	*p*-value
Age - years	60.6 ± 16.5	58.2 ± 16.5	58.2 ± 13.8	0.004
Weight - kg	98.8 ± 33.8	102.2 ± 32.4	91.3 ± 21.0	0.22
Female - %	1670 (54.3%)	375 (36.0%)	29 (43.9%)	< 0.001

Legend: Shown are the characteristics of individuals with reports of adverse reactions to the U.S. Food and Drug Administration Adverse Event Reporting System (FAERS) attributed to ultrasound enhancing agents (UEAs) by UEA brand. Means and standard deviations are provided for continuous variables and compared across brands using analysis of variance. Additionally, the number and percentage of reports in females is compared across brands using a Chi-squared test. One adverse event report attributed to multiple UEAs was excluded

### Rates of serious adverse events

**Fig. 3:**
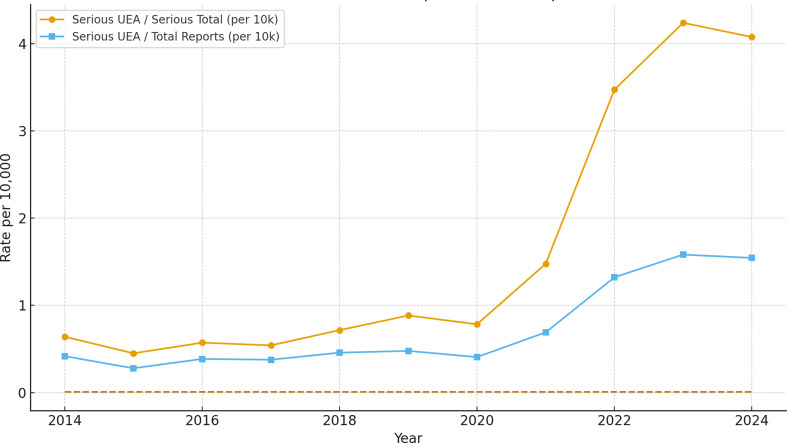
Serious adverse event rates to ultrasound enhancing agents as a function of total and serious adverse event reports to FAERS. Legend: Shown is a line graph demonstrating annual trends in the rate of serious adverse events to ultrasound enhancing agents (UEAs) reported to the U.S. Food and Drug Administration Adverse Event Reporting System (FAERS) from 2014–2024 normalized to overall adverse reports (blue line; squares) and serious adverse events (orange line; circles) reported to FAERS during the same period. Rates in the y-axis are listed as per 10,000 reports

SAE rates to UEAs normalized to total SAEs reported in FAERS increased from a low of 0.45 events/10,000 SAEs in 2015 to a maximum of 4.24 events/10,000 SAEs in 2023 (Fig. 3; Table 4). SAE rates to UEAs normalized to total events reported to FAERS demonstrated similar patterns with a nadir of 0.28 events/10,000 reports in 2015 to a maximum of 1.58 events/10,000 reports in 2023. Compared to 2014 as reference, the relative risk for both SAEs to UEAs as a function of total SAEs as well as SAEs to UEAs normalized to overall reports reported to FAERS significantly increased starting in 2021 with RRs in the range of 1.66–6.62 (Supplemental Table 1, all *p* < 0.05).

**Table 4:** Rates of serious adverse reactions and deaths to ultrasound enhancing agents by year in the FAERS database

Year	Serious Reports for UEAs / Serious Reports	Serious Reports for UEAs / Total Reports	Deaths for UEAs / Total Deaths	Deaths for UEAs / Total Reports
2014	0.64	0.42	0.09	0.008
2015	0.45	0.28	0.34	0.03
2016	0.57	0.39	0.18	0.02
2017	0.54	0.38	0.23	0.02
2018	0.72	0.46	0.48	0.04
2019	0.88	0.48	0.62	0.06
2020	0.78	0.41	0.29	0.02
2021	1.48	0.69	0.56	0.05
2022	3.47	1.32	0.86	0.06
2023	4.24	1.58	1.34	0.09
2024	4.08	1.54	0.61	0.04

Legend: Shown are rates of serious adverse events and deaths attributed to ultrasound enhancing agents (UEAs) and reported to U.S. Food and Drug Administration Adverse Event Reporting System (FAERS) from 2014–2024 by year. Serious adverse events attributed to (UEAs) are indexed to the overall number of serious adverse events reports to FAERS as well as the total number of adverse event reports. Deaths attributed to UEAs are indexed to the total number of deaths reported to FAERS as well as the total number of adverse event reports. Rates are all provided per 10,000 reports

### Differential adverse events across brands

**Fig. 4:**
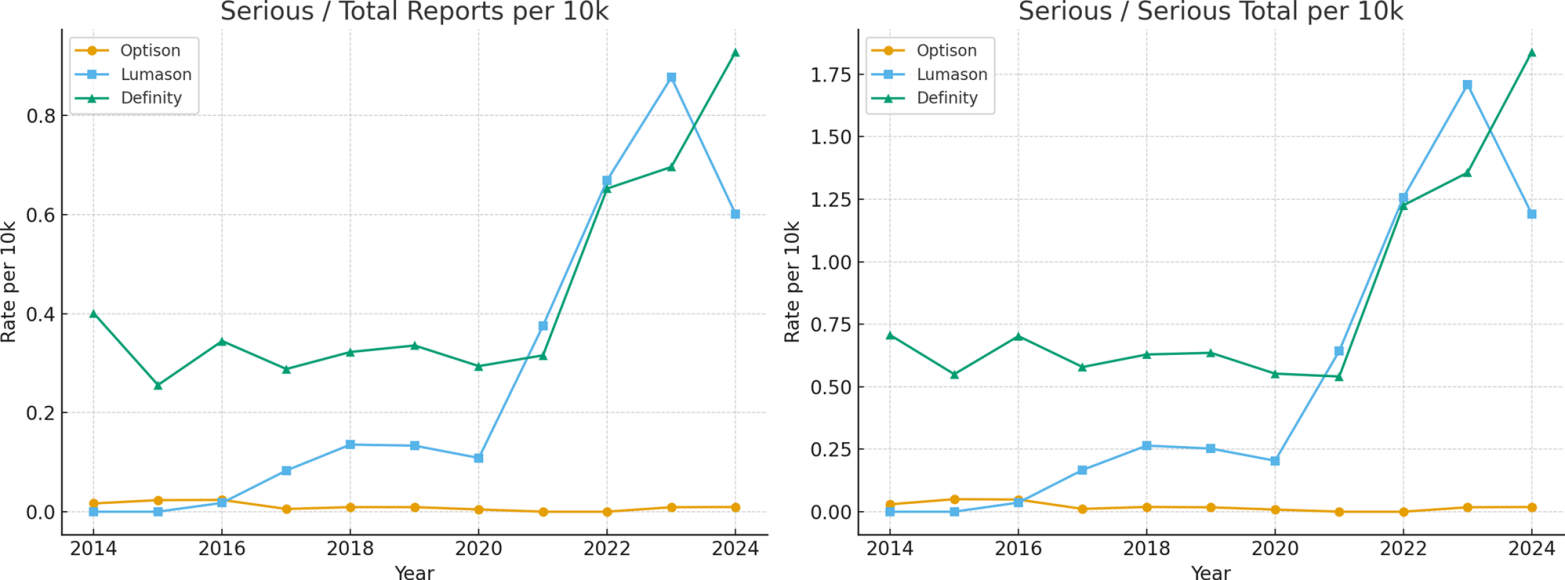
Rates of serious adverse events to ultrasound enhancing agents by brand. Legend: Shown are line graphs demonstrating annual trends in the rates of serious adverse events to ultrasound enhancing agents (UEAs) reported to the U.S. Food and Drug Administration Adverse Event Reporting System (FAERS) from 2014–2024 normalized to overall adverse reports (left panel) and serious adverse events (right panel) reported to FAERS during the same period. Results are stratified by UEA brand, including Optison™ (orange line; circles), Lumason™ (blue line; squares), and Definity™ (green line; triangles). Rates in the y-axis are listed as per 10,000 reports

Of those with AEs to Definity™ (*N* = 3076), 987 (32.1%) were serious and 44 (1.4%) resulted in death (Supplemental Table 2). Of those with AEs to Lumason™ (*N* = 1042), 667 (64.0%) were serious and 46 (4.4%) resulted in death. Of those with AEs to Optison™ (*N* = 66), 18 (27.3%) were serious and 2 (3.0%) resulted in death. While the number of AEs followed similar temporal trends as UEAs as a class across study years (Supplemental Tables 3–5), when considering SAEs as a rate (i.e. as a function of the total number of AE reports to FAERS), rates of SAEs were overall low (< 1:10,000) regardless of brand and not statistically different over time (p-value for all trend tests > 0.05). However, despite low absolute rates of SAEs across the therapeutic class, Lumason™ and Definity™ both demonstrated an increase in SAE rates (relative to baseline values) in 2020 before declining in 2024 for Lumason™ (Fig. 4, Supplemental Tables 6–11). Either before 2020 (Definity™ vs. Lumason™, RR 2.81, 95% CI 0.24–32.5, *p* = 0.41) or after 2020 (Definity™ vs. Lumason™, RR 1.08, 95% CI 0.23–5.03, *p* = 0.92), there were no significant differences in SAE rates between these two agents (all *p* > 0.05).

### Relative contribution of reporting

**Fig. 5:**
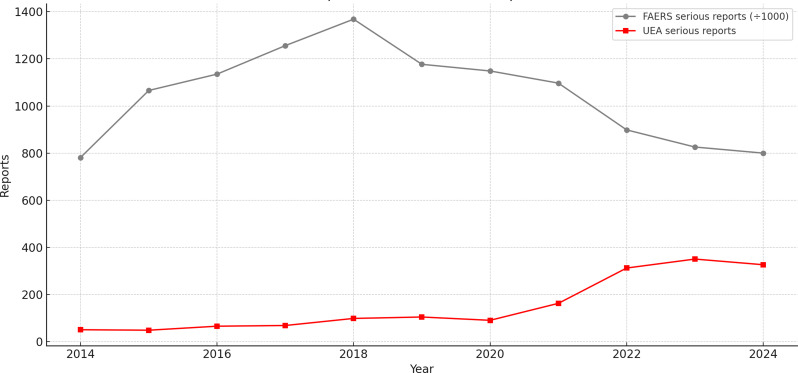
Numbers of reported serious adverse events to ultrasound enhancing agents compared to overall serious adverse events in FAERS, 2014–2024. Legend: Shown is a line graph demonstrating the number of adverse events to ultrasound enhancing agents (UEAs; red line; squares) relative to overall serious adverse events (gray line; circle) reported to the U.S. Food and Drug Administration Adverse Event Reporting System (FAERS) from 2014–2024. Overall serious adverse events are reported on the y-axis as per 1,000 for scaling purposes

This relative increase in SAEs observed for Lumason™ and Definity™ can be only partially attributed to declining reports of non-UEA related SAEs and deaths (normalized to total reports) after 2018 (Fig. 5; Supplemental Fig. 1). The observed increase in SAEs to UEAs from 2014 (*N* = 50) to 2023 (*N* = 350) was 300. During the same time period, SAEs increased in FAERS from 678,953 to 1,135,192 representing a growth rate of 67.2%. The expected SAE rate in 2023 due to background reporting alone would accordingly be 50 × 1.672 = 83.6, thus the expected increase would be 33.6 (i.e. 83.6–50). Accordingly, the proportion attributable to background reporting is 33.6/300 = 11.2%.

### Comparison to other contrast agents and PEGylated agents

**Fig. 6:**
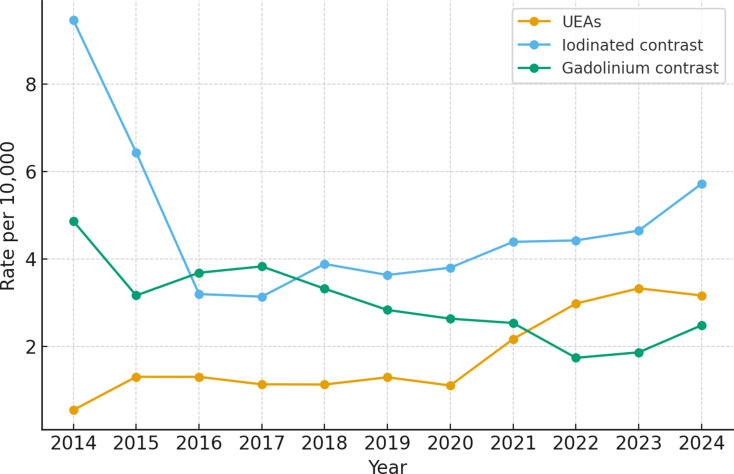
Rates of total adverse events to ultrasound enhancing agents, iodinated contrast, and gadolinium contrast in FAERS, 2014–2024. Legend: Shown are line graphs demonstrating annual trends in the rates of serious adverse events to ultrasound enhancing agents (UEAs; orange line), iodinated contrast (blue line), and gadolinium contrast (green line) reported to the U.S. Food and Drug Administration Adverse Event Reporting System (FAERS) from 2014–2024 normalized to overall adverse reports reported to FAERS during the same period. Rates in the y-axis are listed as per 10,000 reports. As rates of adverse reactions to pegfilgrastim (Neulasta™) are significantly higher, they were not included on this chart

From 2014 to 2024, total reports to FAERS for UEAs as class increased by from 0.54 per 10,000 to 3.16 per 10,000 (average change, + 16.7%/year, 95% CI 15.4–18.0%/year) compared to a decrease from 9.46 per 10,000 to 5.72 per 10,000 for iodinated contrast (average change, -2.1%/year, 95% CI -2.7% to -1.5%/year) and a decrease from 4.86 per 10,000 to 2.48 per 10,000 for gadolinium contrast (average change, -7.6%/year, 95% CI -8.4% to -6.9%/year). (Fig. 6, Supplemental Tables 12–14). By comparison, total reports to FAERS for pegfilgrastim (i.e. Neulasta™) increased from 17.07 per 10,000 to 20.77 per 10,000 (average change, 2.1%/year, 95% CI 1.9–2.4%/year) (Supplemental Tables 12–14). Compared to UEAs, both iodinated contrast (RR 2.47, 95% CI 2.38–2.56, *p* < 0.001) and gadolinium contrast (RR 1.54, 95% CI 1.48–1.60, *p* < 0.001) were associated substantially greater AE risk on average across years.

### External comparison

Across Piedmont Health System, 417,847 TTEs were performed from 2021 to 2024, of which 181,759 (43.5%) received UEAs (171,346 [94.3%] Lumason™ and 10,413 [5.7%] Optison™). A total of 39 serious AEs (mean 2.07 ± 1.4 SAEs per 10,000 UEA administrations) and 60 non-serious AEs (mean 3.14 ± 1.23 AEs per 10,000 UEA administrations) occurred over the 4 years of study, all of which were observed in patients receiving Lumason™. AE rates peaked in 2023 before declining in 2024, similar to national patterns (Supplemental Fig. 2; Supplemental Table 15).

## Discussion

### Summary of findings

In this critical re-analysis of the publicly available FDA FAERS dataset, 2014–2024, we identified that 11.2% of the observed increase in reported SAEs to UEAs can be attributed to declining SAE reporting from 2021 to 2024. While a small observed risk of SAEs to UEAs was observed, limited to the two commercially available PEGylated microbubbles (e.g. Lumason™ and Definity™), there were no differences in reaction rates observed between these two UEA brands, and differences in the utilization patterns, reporting bias, and temporal confounding make it difficult to draw definitive conclusions about brand-related differences. Despite this, the observed absolute rate of SAEs to UEAs remains among the safest of all contrast media, with overall AE risks substantially below gadolinium and iodinated contrast during the same period, overall supporting their continued safety as a class. While these results overall support the safety of UEAs, they suggest the need for adoption of preparedness strategies and better screening tools to identify patients at risk for reactions to reduce risks further.

### Pertinent background

While UEAs are historically one of the safest forms of contrast media across several decades of investigation [5–7, 16–20], use of UEAs carries a 1:10,000 to 1:15,000 risk of a severe anaphylactoid reaction, felt to be predomimantly to CARPA reactions [8]. As such, laboratory policies for safe and timely responses to possible reactions have been made a requirement for accreditation [21] and a key component of professional societal guidelines on UEA use [2, 22–24]. However, in 2020–2022, during the mass vaccination campaign for COVID-19, reports of anaphylaxis to the mRNA vaccine [25, 26] drew international attention, with some speculating a causative role for PEG [25–28], a component of the mRNA lipid nanoparticles and a common ingredient in numerous cosmetics, processed foods, radiopharmaceuticals, and in agricultural or industrial manufacturing [29]. Previously, reports of allergy to PEG were exceedingly rare [28, 30] with naturally occurring antibodies to PEG in historical studies occurring in 0.2% of healthy blood donors [31]. However, more contemporary studies have reported a higher incidence of anti-PEG antibodies ranging from 5 to 9% of the general population [32] leading to concerns that widespread use of PEG in household products resulted in increased immune exposure [33]. Moreover, PEG-2000 (i.e. PEG with a molecular weight of 2,000 g/mol) was the only recognized allergen in the COVID-19 vaccine and reported rates of PEG allergies were on the rise [28], leading to conjectures about PEG’s causative role in anaphylaxis to the mRNA vaccines [25, 26, 34]. The majority of these vaccine reactions subsequently turned out to be non-reproducible, non-allergic reactions when tested and re-challenged, and only rarely attributable to PEG in less than 1% of reactors [35–39] with heightened awareness and nocebo effect playing a significant role in overreporting of mRNA vaccine allergy reactions [37].

In this setting, published reports of individuals with PEG allergy demonstrating cross-reactivity to PEGylated UEAs [27, 28, 40, 41] led to further speculation about the role of the COVID-19 mass vaccination campaign in sensitizing individuals to intravenously administered PEGylated agents. Of FDA-approved UEAs, PEG-5000 is incorporated in the lipid monolayer of Definity™ preparations (approximately 0.304 mg/vial) and PEG-4000 is used as an excipient in Lumason™ preparations (approximately 24.56 mg/vial) [42]. In response, *MedWatch*, the FDA’s medical product safety reporting program for professionals, patients, and consumers issued a report about presumed Type 1 hypersensitivity reactions to UEAs due to PEG [42]. While this report was based on 11 cases over 10 years (with an estimated rate of reaction < 1 per million doses) during which time the majority of the historical data demonstrating safety of UEAs were published [5–7, 16–20, 42], its publication led to changes in labeling with the addition of new contraindications for those with known hypersensitivity to PEG or bowel preparations (which frequently contain high molecular weight PEG) [42]. A subsequent multi-center retrospective study across 4 large health systems [11] performing contrast-enhanced echocardiography suggested an increase in SAEs to UEAs in contemporary practice, particularly in those who received Lumason™ and those with prior receipt of the Moderna™ COVID-19 vaccine. However, given contemporary use of Lumason™ was compared to historical use of Definity™, it was previously unclear whether this brand-specific effect could be due to changes in temporal trends in UEA safety. Moreover, in a subsequent large 11.4 million patient claims study [14] evaluating patients who received UEAs for transthoracic or stress echocardiography, 2018–2022, after accounting for differences in clinical conditions between individuals who received and didn’t receive UEAs, use of UEAs was associated with decreased mortality within 48 h of use, similar across brands and years of study and akin to results from historical safety studies [6] and studies evaluating safety in the radiology literature [43], leading to confusion in how to reconcile such results.

### Interpretation and hypotheses

In this analysis of FDA publicly reported data, we observed an increase in AEs as a function of overall reports starting in 2021, peaking in 2023 and declining thereafter. This pattern in UEAs mirrors overall trends in reported AEs to FAERS which peaked in 2022 during the height of the COVID-19 pandemic before declining. This pattern also mirrors data from echocardiography laboratories across Piedmont Health System, a large integrated delivery network across Georgia with peak AEs observed in 2023 before declining. We did not observe a similar secular increased rate of reaction to other contrast media such as iodinated and gadolinium contrast, both of which had declining risks during the same period. The vast majority of AEs to UEAs were nonserious, with < 20 annual deaths despite widespread use of UEAs across the US during this time and significantly lower than the AE rate to iodinated and gadolinium contrast, overall suggesting continued safety of UEAs as a class. As a declining denominator (i.e. overall AEs reported to FAERS) could theoretically contribute to the appearance of a relative increase in AEs to UEAs without an increase in the numerator (i.e. SAEs to UEAs), we identify that this reporting effect explains 11.2% of the increase in AEs observed, with the majority not explained by reporting.

Reasons for this apparent rise and fall in SAEs to UEAs remain unclear, particularly given total AEs reported to FAERS began increasing prior to the COVID-19 pandemic [28]. Accordingly, such data should be viewed with a critical lens. As FAERS relies on self-report, does not account for duplicate entries, lacks information on the denominator of overall use of UEAs during this time period, and events are not clinically adjudicated, it is likely that such findings may be an artifact of reporting behavior and background changes in utilization given prior safety data supporting broad safety of UEAs as a class with similarities across brands [14]. Furthermore, brand-specific differences, while confined to the two PEGylated agents (i.e. Lumason™ and Definity™), could reflect market penetrance as the two largest distributors of UEAs as well as differential use of Optison™ in outpatient settings [14].

Another possible mechanistic explanation, while speculative at present, could also be a direct effect of COVID-19 on PEG-specific immunity [44], sensitizing certain individuals to PEG [25–28], making them susceptible to allergic events upon re-exposure to PEG, particularly administered parenterally as is the case with UEAs. Supporting this hypothesis is that such increases were not observed in other non-PEGylated forms of contrast such as iodinated and gadolinium contrast but were observed for pegfilgrastim (i.e. Neulasta™), a common PEGylated form of granulocyte colony stimulating factor used for chemotherap. As the molecular weight of PEG is directly related to the degree of immune response [41], it is possible that lower molecular weight PEG as contained in the COVID-19 vaccines (i.e. PEG-2000) was insufficient to directly cause an allergic reaction to the vaccine, but nevertheless sensitized at-risk individuals upon re-exposure at a higher molecular weight [28].

While the mechanism of such reactions to UEAs remains unclear, recently developed standardized laboratory assays to detect anti-PEG antibodies [45] have identified appreciable levels of IgE anti-PEG antibodies in patients with known PEG allergy that are not present in healthy controls, suggesting Type 1 hypersensitivity reactions (as opposed to the more typical CARPA reaction) may be responsible in some cases. However, as CARPA reactions are potentially facilitated in the presence of IgG and IgM sensitization to PEG [46], and these antibody types were measured to occur in a pre-pandemic random population sample at a rate of 5–9% and 3–6%, respectively [32], it is possible that potentiation of CARPA may be the predominant mechanism for the observed increased risk to UEAs. By comparison, the rate of anti-PEG IgE sensitization in the same sample was around 0.2%, and clinically symptomatic IgE-mediated PEG allergy is estimated by experts to be in the range of one per million [28].

### Implications for practice

Despite a possible mechanistic pathway for PEG-sensitization to explain some of the observed findings, it is difficult at present to attribute such findings solely to PEG given the challenges inherent in interpreting FAERS data. Furthermore, the observed increase in SAEs preceded the COVID-19 pandemic [28] arguing against a PEG-specific effect. Given these challenges, what additional insights can be gleaned from such data? First, our results suggest large differences in reporting of AEs during the COVID-19 pandemic, such that the brand-specificity observed in the prior publication by Ali et al. [11] may have been due to the time period in which reactions were evaluated (i.e. pre-COVID for Definity™ and during-COVID for Lumason™).

Second, these findings reinforce the overall safety of UEAs as a class, and in the context of recent safety studies [14], suggest that the continued benefit of UEA use outweighs the risks of non-use. Given the lower rate of events to SAEs than gadolinium and iodinated contrast during the same time period, the fundamental safety of UEAs as a class is upheld, raising questions about whether the FDA’s black box warning on UEAs (but not on gadolinium or iodinated contrast), should be removed in the absence of data showing a higher risk than these agents and presence of data suggesting the opposite.

Third, while reactions were uncommon in UEAs, the known side effect profile suggests the need for broad vigilance and preparedness for AEs among settings where UEA administration is occurring, including cardiac, vascular, and general ultrasound laboratories. To this end, in collaboration with 14 societies representing ultrasound professionals across the globe, we have recently published consensus guidelines [22] on safe laboratory practice to standardize approaches towards timely recognition and treatment of such reactions. Fourth, further investigation is needed to evaluate the impact of PEG antibodies and their role in potentiating CARPA reactions. While of uncertain significance broadly, PEG may be of importance in a small minority of patients, for which better screening mechanisms are needed. In particular, prior reactions to PEGylated agents other than bowel preparations (e.g. depo-steroids) may be overlooked as being related to PEG [28], thus unnecessarily exposing patients to potential increased risks without such a screen. Further research is needed on the clinical impact of screening for PEG and ways to identify individuals at-risk for PEG-mediated potentiation of CARPA. Despite these considerations, our data reinforce that reactions to PEG remain uncommon and should not dissuade appropriate use of UEAs or other PEG-containing substances, many of which provide significant diagnostic and therapeutic benefit [22, 42].

### Limitations

Several limitations should be considered when interpreting these results. First, as FAERS relies on clinician, industry, or patient self-reporting of AEs, it is possible that events may be underreported, though this underreporting is likely nondifferential and thus should not influence overall observed trends. Second, as the denominator of total doses of UEAs administered during the study period (overall and by brand) is unknown, it is not possible to estimate a reliable rate of reaction. As such, reported rates (indexed to total number of AEs or SAEs) represent approximations. Third, dynamic changes to AE reporting and healthcare utilization during COVID-19 [47] may have influenced the observed trends in ways that are not readily quantifiable. Fourth, FAERS does not contain reliable information on the indications for imaging, UEA use, or the clinical comorbidities associated with those receiving UEAs, and thus it is not possible to exclude residual confounding in the observed results. Fifth, it is possible that certain individuals may have multiple reported reactions in FAERS as unique patient identifiers are not available. Nevertheless, given the infrequency of SAEs to UEAs, it is most likely that repeat events in FAERS are relatively rare and thus the analysis assumes no significant clustering of outcomes by individual. As stated in the methods section, deaths may be counted as SAEs as well and thus the rate of SAEs reflects both fatal and non-fatal events. Sixth, FAERS does not allow analysis of whether specific use (e.g. cardiac vs. body use) is associated with different rates of SAEs. However, prior studies [43] have reported similar event rates across applications, making this unlikely. Seventh, analyses of Piedmont data, while supportive, is nevertheless limited to a single healthcare system with limited use of UEAs beyond Lumason™, and therefore could reflect changes in utilization rather than a brand-specific effect.

## Conclusions

In a re-analysis of publicly reported FDA safety data from 2014 to 2024, 11.2% of the observed increase in reported SAEs to UEAs is attributable to reporting changes. Given large-scale changes in reporting during this period, however, brand-specific comparisons should be interpreted with caution. Overall, SAEs to UEAs remain exceedingly uncommon and rates are declining, broadly supporting continued use, but suggest the need for rigorous and standardized safety protocols and further investigation into the mechanisms of observed allergic reactions.

## Supplementary Information

Below is the link to the electronic supplementary material.

Supplementary Material 1

## Data Availability

Data used for the study are publicly available through the FDA FAERS website.

## Abbreviations

AE: Adverse event
CARPA: Complement Activation Related Pseudoallergy
CI: Confidence interval
FAERS: Food and Drug Administration Adverse Event Reporting System
FDA: Food and Drug Administration
PEG: Polyethylene glycol
RR: Rate ratio
SAEs: Serious adverse events
UEA: Ultrasound Enhancing Agents
