# Supplementary material for: Reported safety events from ultrasound enhancing agents: a critical reappraisal
Source: Echo Res Pract. 2026 Jul 13;13:25. doi: 10.1186/s44156-026-00127-1 (PMC13360243; doi:10.1186/s44156-026-00127-1)
Supplement: Supplementary file 1 — Supplementary Material 1 [file 44156_2026_127_MOESM1_ESM.pdf]

Supplemental Appendix

Supplemental Figure 1: Numbers of Reported Deaths Attributed to Ultrasound Enhancing Agents Compared to Overall Deaths Reported to FAERS, 2014-2024

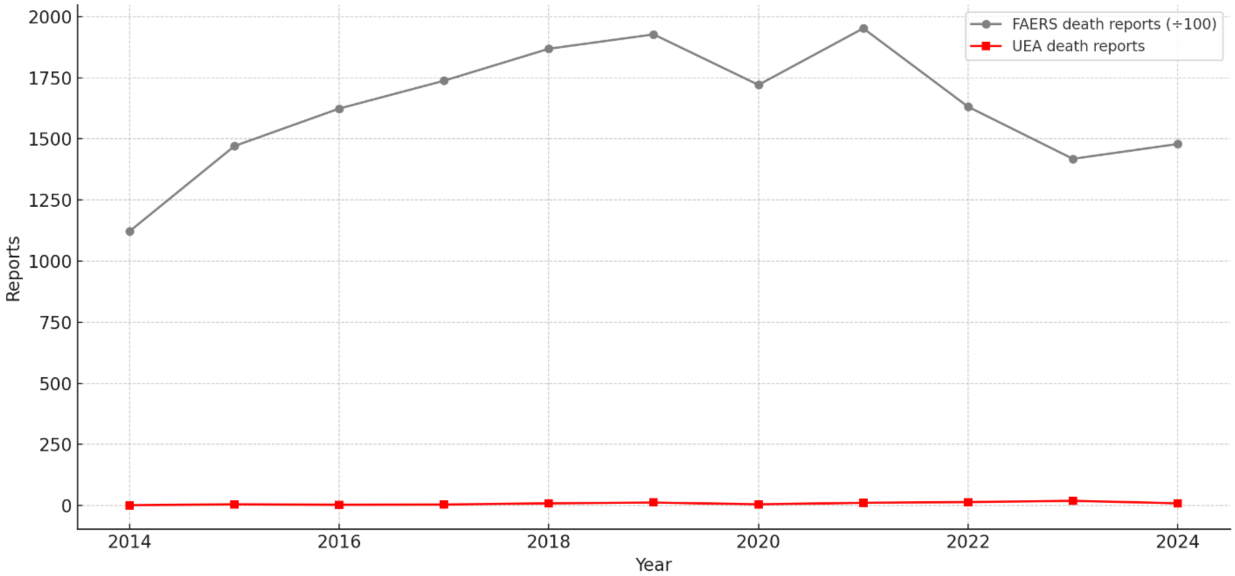

Legend: Shown is a line graph demonstrating the number of deaths attributed to ultrasound enhancing agents (UEAs; red line; squares) relative to overall deaths (gray line; circle) reported to the U.S. Food and Drug Administration Adverse Event Reporting System (FAERS) from 2014-2024. Overall death events are reported on the y-axis as per 100 for scaling purposes.

Supplemental Figure 2: Adverse Event Rates to Ultrasound Enhancing Agents Across the Piedmont Health System

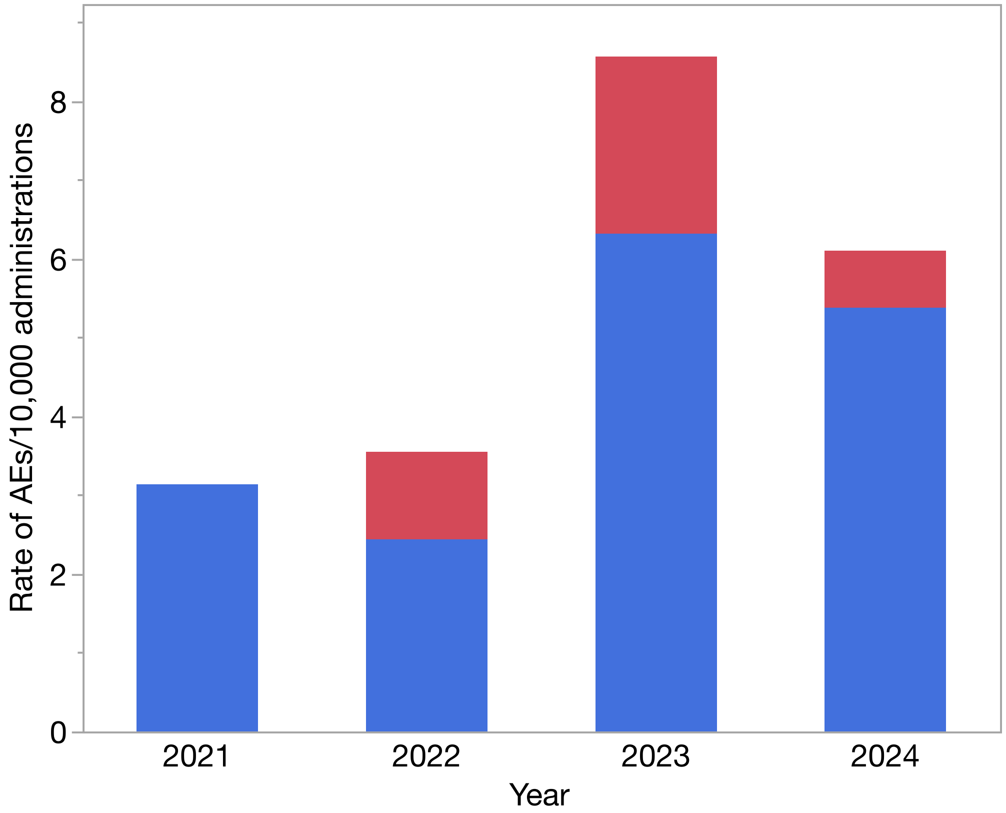

Legend: Shown is a stacked bar graph displaying the rates of serious (red) and non-serious (blue) adverse events (AEs) per 10,000 UEA administrations (y-axis) across the Piedmont Health System in Georgia by year, overall mirroring national trends.

Supplemental Table 1: Relative Risk of Serious Adverse Events to Ultrasound Enhancing Agents Reported to FAERS by Year

**Serious adverse events to UEAs per *total* adverse events reported to FAERS**			
**Year**	**Rate Ratio**	**95% CI**	**p-value**
2014	Ref	Ref	Ref
2015	0.67	0.45 – 0.99	0.05
2016	0.93	0.64 – 1.34	0.68
2017	0.90	0.63 – 1.30	0.58
2018	1.10	0.78 – 1.54	0.59
2019	1.15	0.82 – 1.61	0.43
2020	0.98	0.69 – 1.38	0.89
2021	**1.66**	**1.21 – 2.27**	**0.002**
2022	**3.17**	**2.35 – 4.27**	**< 0.001**
2023	**3.79**	**2.82 – 5.10**	**< 0.001**
2024	**3.70**	**2.75 – 4.98**	**< 0.001**
**Serious adverse events to UEAs per *serious* adverse events reported to FAERS**			
**Year**	**Rate Ratio**	**95% CI**	**p-value**
2014	Ref	Ref	Ref
2015	0.70	0.47 – 1.05	0.08
2016	0.89	0.62 – 1.29	0.55
2017	0.85	0.59 – 1.22	0.37
2018	1.12	0.80 – 1.57	0.52
2019	1.38	0.99 – 1.93	0.06
2020	1.22	0.87 – 1.73	0.25
2021	**2.31**	**1.68 – 3.17**	**< 0.001**
2022	**5.43**	**4.03 – 7.31**	**< 0.001**
2023	**6.62**	**4.92 – 8.91**	**< 0.001**
2024	**6.37**	**4.73 – 8.58**	**< 0.001**

Legend: Shown are the rate ratios, 95% confidence intervals (CIs), and Wald p-values for the relative risk of serious adverse events attributed to ultrasound enhancing agents (UEAs) reported to the U.S. Food and Drug Administration Adverse Event Reporting System (FAERS) from 2014-2024 by year. Results are derived from Poisson regression using 2014 as the reference (Ref) year. The top panel indicates the rate ratio for serious adverse events attributed to UEAs as a function of total adverse events reported to FAERS. The bottom panel indicates the rate ratio for serious adverse events attributed to UEAs as a function of overall serious adverse events reported to FAERS. Statistically significant results at a $p < 0.05$ threshold are bolded.

Supplemental Table 2: Number of Adverse Events Reported to FAERS, 2014-2024, by Adverse Event Severity and Ultrasound Enhancing Agent Brand

	**Total Reports (N = 4185)**	**Serious Reports (N = 1673)**	**Non-Serious (N = 2420)**	**Deaths (N = 92)**
Definity™	3076	987	2045	44
Lumason™	1042	667	329	46
Optison™	66	18	46	2
Multiple	1	1	0	0

Legend: Shown are the total number of adverse event reports, serious adverse reports, non-serious adverse event reports, and adverse events resulting in death reported to the U.S. Food and Drug Administration Adverse Event Reporting System (FAERS) and attributed to ultrasound enhancing agents (UEAs) from 2014-2024 by brand of UEA. One individual had a serious adverse event attributed to multiple agents. N = number of reports.

Supplemental Table 3: Number of Adverse Event Reports in FAERS, 2014-2024, Attributed to Optison™ by Year and Severity of Reaction

Year	Total Reports (N = 64)	Serious Reports (N = 18)	Non-Serious Reports (N = 46)	Deaths (N = 2)
2014	10	2	8	0
2015	6	4	2	0
2016	4	4	0	0
2017	5	1	4	0
2018	4	0	4	0
2019	5	2	3	1
2020	1	1	0	0
2021	3	0	3	0
2022	5	0	5	0
2023	12	2	10	0
2024	9	2	7	1

Legend: Shown are the total number of adverse event reports, serious adverse reports, non-serious adverse event reports, and adverse events resulting in death reported to the U.S. Food and Drug Administration Adverse Event Reporting System (FAERS) and attributed to Optison™ from 2014-2024 by year. N = number of reports.

Supplemental Table 4: Number of Adverse Event Reports in FAERS, 2016-2024, Attributed to Lumason™ by Year and Severity of Reaction

Year	Total Reports (N = 996)	Serious Reports (N = 667)	Non-Serious Reports (N = 329)	Deaths (N = 46)
2016	3	3	0	0
2017	19	15	4	2
2018	45	29	16	3
2019	58	29	29	5
2020	40	24	16	2
2021	117	88	29	7
2022	225	158	67	9
2023	317	194	123	13
2024	172	127	45	5

Legend: Shown are the total number of adverse event reports, serious adverse reports, non-serious adverse event reports, and adverse events resulting in death reported to the U.S. Food and Drug Administration Adverse Event Reporting System (FAERS) and attributed to Lumason™ from 2016-2024 by year. 2016 was used as the starting year given FDA approval for Lumason™ in 2016. N = number of reports.

Supplemental Table 5: Number of Adverse Event Reports in FAERS, 2016-2024, Attributed to Definity™ by Year and Severity of Reaction

Year	Total Reports (N = 3032)	Serious Reports (N = 987)	Non-Serious Reports (N = 2045)	Deaths (N = 44)
2014	55	48	7	1
2015	218	44	174	5
2016	212	58	154	3
2017	180	52	128	2
2018	192	69	123	6
2019	218	73	145	6
2020	203	65	138	3
2021	388	74	314	4
2022	473	154	319	5
2023	407	154	253	6
2024	486	196	290	3

Legend: Shown are the total number of adverse event reports, serious adverse reports, non-serious adverse event reports, and adverse events resulting in death reported to the U.S. Food and Drug Administration Adverse Event Reporting System (FAERS) and attributed to Definity™ from 2014-2024 by year. N = number of reports.

Supplemental Table 6: Rates of Serious Adverse Reactions and Deaths to Optison™ by Year in the FAERS Database

**Year**	**Serious Reports for Optison™ / *Serious* Reports**	**Serious Reports for Optison™/ *Total* Reports**	**Deaths for Optison™/ Total *Deaths***	**Deaths for Optison™/ Total *Reports***
2014	0.03	0.02	0.00	0.00
2015	0.05	0.02	0.00	0.00
2016	0.05	0.02	0.00	0.00
2017	0.01	0.01	0.00	0.00
2018	0.00	0.00	0.00	0.00
2019	0.02	0.01	0.06	0.005
2020	0.01	0.005	0.00	0.00
2021	0.00	0.00	0.00	0.00
2022	0.00	0.00	0.00	0.00
2023	0.02	0.01	0.00	0.00
2024	0.02	0.01	0.07	0.005
p-value for trend	*0.82*	*0.87*	*0.78*	*0.94*

Legend: Shown are rates of serious adverse events and deaths attributed to Optison™ and reported to U.S. Food and Drug Administration Adverse Event Reporting System (FAERS) from 2014-2024 by year. Serious adverse events attributed to Optison™ are indexed to the overall number of serious adverse events reports to FAERS as well as the total number of adverse event reports. Deaths attributed to Optison™ are indexed to the total number of deaths reported to FAERS as well as the total number of adverse event reports. Rates are all provided per 10,000 reports. Additionally provided are p-values for Poisson trend tests across years.

Supplemental Table 7: Relative Risk of Serious Adverse Events to Optison™ Reported to FAERS by Year

Serious adverse events to Optison™ per *total* adverse events reported to FAERS			
**Year**	**Rate Ratio**	**95% CI***	**p-value**
2014	Ref	Ref	Ref
2015	1.4	0.33 – 5.90	0.65
2016	1.43	0.34 – 6.01	0.63
2017	0.33	0.04 – 2.77	0.31
2018	0.25	0.03 – 2.14	0.21
2019	0.55	0.11 – 2.73	0.46
2020	0.27	0.03 – 2.31	0.23
2021	0.25	0.03 – 2.14	0.21
2022	0.25	0.03 – 2.14	0.21
2023	0.54	0.11 – 2.71	0.45
2024	0.57	0.12 – 2.86	0.49
**Serious adverse events to Optison™ per *serious* adverse events reported to FAERS**			
**Year**	**Rate Ratio**	**95% CI**	**p-value**
2014	Ref	Ref	Ref
2015	1.63	0.38 – 6.93	0.51
2016	1.55	0.36 – 6.64	0.56
2017	0.35	0.04 – 2.97	0.34
2018	0.24	0.03 – 2.08	0.2
2019	0.59	0.12 – 2.84	0.51
2020	0.29	0.03 – 2.41	0.25
2021	0.24	0.03 – 2.07	0.2
2022	0.24	0.03 – 2.07	0.2
2023	0.6	0.12 – 2.87	0.52
2024	0.64	0.13 – 3.05	0.57

Legend: Shown are the rate ratios, 95% confidence intervals (CIs), and Wald p-values for the relative risk of serious adverse events attributed to Optison™ reported to the U.S. Food and Drug Administration Adverse Event Reporting System (FAERS) from 2014-2024 by year. Results are derived from Poisson regression using 2014 as the reference (Ref) year. The top panel indicates the rate ratio for serious adverse events attributed to Optison™ as a function of total adverse events reported to FAERS. The bottom panel indicates the rate ratio for serious adverse events attributed to Optison™ as a function of overall serious adverse events reported to FAERS. *Confidence intervals are calculated using the 0.5 continuity correction to ensure finite intervals are reported.

Supplemental Table 8: Rates of Serious Adverse Reactions and Deaths to Lumason™ by Year in the FAERS Database

**Year**	**Serious Reports for Lumason™ / *Serious* Reports**	**Serious Reports for Lumason™/ *Total* Reports**	**Deaths for Lumason™/ Total *Deaths***	**Deaths for Lumason™/ Total *Reports***
2016	0.04	0.02	0.00	0.00
2017	0.17	0.08	0.12	0.01
2018	0.26	0.14	0.15	0.01
2019	0.25	0.13	0.29	0.02
2020	0.20	0.11	0.10	0.01
2021	0.64	0.38	0.37	0.03
2022	1.26	0.67	0.52	0.04
2023	1.71	0.88	0.80	0.06
2024	1.19	0.60	0.34	0.02
p-value for trend	*0.08*	*0.21*	*0.34*	*0.81*

Legend: Shown are rates of serious adverse events and deaths attributed to Lumason™ and reported to U.S. Food and Drug Administration Adverse Event Reporting System (FAERS) from 2016-2024 by year. Serious adverse events attributed to Lumason™ are indexed to the overall number of serious adverse events reports to FAERS as well as the total number of adverse event reports. Deaths attributed to Lumason™ are indexed to the total number of deaths reported to FAERS as well as the total number of adverse event reports. Rates are all provided per 10,000 reports. Additionally provided are p-values for Poisson trend tests across years. Only years 2016-2024 are considered given approval of Lumason™ in 2016.

Supplemental Table 9: Relative Risk of Serious Adverse Events to Lumason™ Reported to FAERS by Year

**Serious adverse events to Lumason™ per *total* adverse events reported to FAERS**			
**Year**	**Rate Ratio**	**95% CI***	**p-value**
2016	Ref	Ref	Ref
2017	4.67	1.39 – 15.7	0.013
2018	7.61	2.42 – 23.9	<0.001
2019	7.49	2.38 – 23.6	<0.001
2020	6.09	1.92 – 19.3	0.002
2021	21.1	7.34 – 60.8	<0.0001
2022	37.7	14.2 – 100.3	<0.0001
2023	49.2	18.6 – 130.0	<0.0001
2024	33.8	12.7 – 90.3	<0.0001
**Serious adverse events to Lumason™ per *serious* adverse events reported to FAERS**			
**Year**	**Rate Ratio**	**95% CI**	**p-value**
2016	Ref	Ref	Ref
2017	4.6	1.45 – 14.6	0.01
2018	7.28	2.40 – 22.1	<0.001
2019	6.95	2.29 – 21.0	<0.001
2020	5.62	1.84 – 17.2	0.002
2021	17.7	6.28 – 49.9	<0.0001
2022	34.8	13.2 – 91.4	<0.0001
2023	47.2	18.0 – 123.9	<0.0001
2024	32.9	12.5 – 86.6	<0.0001

Legend: Shown are the rate ratios, 95% confidence intervals (CIs), and Wald p-values for the relative risk of serious adverse events attributed to Lumason™ reported to the U.S. Food and Drug Administration Adverse Event Reporting System (FAERS) from 2016-2024 by year. Results are derived from Poisson regression using 2016 as the reference (Ref) year as the first year of Lumason™ approval. The top panel indicates the rate ratio for serious adverse events attributed to Lumason™ as a function of total adverse events reported to FAERS. The bottom panel indicates the rate ratio for serious adverse events attributed to Lumason™ as a function of overall serious adverse events reported to FAERS. *Confidence intervals are calculated using the 0.5 continuity correction to ensure finite intervals are reported.

Supplemental Table 10: Rates of Serious Adverse Reactions and Deaths to Definity™ by Year in the FAERS Database

**Year**	**Serious Reports for Definity™ / *Serious* Reports**	**Serious Reports for Definity™/ *Total* Reports**	**Deaths for Definity™/ Total *Deaths***	**Deaths for Definity™/ Total *Reports***
2014	0.71	0.40	0.08	0.01
2015	0.55	0.26	0.34	0.03
2016	0.7	0.34	0.21	0.02
2017	0.58	0.29	0.12	0.01
2018	0.63	0.32	0.31	0.03
2019	0.64	0.34	0.35	0.03
2020	0.55	0.29	0.16	0.01
2021	0.54	0.32	0.21	0.02
2022	1.23	0.65	0.29	0.02
2023	1.36	0.70	0.37	0.03
2024	1.84	0.93	0.20	0.01
p-value for trend	*0.29*	*0.45*	*0.85*	*0.98*

Legend: Shown are rates of serious adverse events and deaths attributed to Definity™ and reported to U.S. Food and Drug Administration Adverse Event Reporting System (FAERS) from 2014-2024 by year. Serious adverse events attributed to Definity™ are indexed to the overall number of serious adverse events reports to FAERS as well as the total number of adverse event reports. Deaths attributed to Definity™ are indexed to the total number of deaths reported to FAERS as well as the total number of adverse event reports. Rates are all provided per 10,000 reports. Additionally provided are p-values for Poisson trend tests across years.

Supplemental Table 11: Relative Risk of Serious Adverse Events to Definity™ Reported to FAERS by Year

**Serious adverse events to Definity™ per *total* adverse events reported to FAERS**			
**Year**	**Rate Ratio**	**95% CI***	**p-value**
2014	Ref	Ref	Ref
2015	0.64	0.27 – 1.53	0.31
2016	0.86	0.39 – 1.88	0.71
2017	0.72	0.32 – 1.63	0.44
2018	0.81	0.36 – 1.81	0.6
2019	0.84	0.38 – 1.85	0.65
2020	0.73	0.32 – 1.64	0.45
2021	0.79	0.35 – 1.75	0.56
2022	1.63	0.82 – 3.26	0.16
2023	1.74	0.88 – 3.45	0.11
2024	2.32	1.20 – 4.50	0.012
**Serious adverse events to Definity™ per *serious* adverse events reported to FAERS**			
**Year**	**Rate Ratio**	**95% CI**	**p-value**
2014	Ref	Ref	Ref
2015	0.78	0.34 – 1.77	0.55
2016	1	0.47 – 2.14	0.99
2017	0.82	0.37 – 1.82	0.63
2018	0.89	0.41 – 1.95	0.76
2019	0.9	0.42 – 1.97	0.79
2020	0.78	0.35 – 1.73	0.55
2021	0.76	0.34 – 1.68	0.51
2022	1.73	0.87 – 3.45	0.11
2023	1.92	0.97 – 3.80	0.06
2024	2.6	1.35 – 5.02	0.004

Legend: Shown are the rate ratios, 95% confidence intervals (CIs), and Wald p-values for the relative risk of serious adverse events attributed to Definity™ reported to the U.S. Food and Drug Administration Adverse Event Reporting System (FAERS) from 2014-2024 by year. Results are derived from Poisson regression using 2014 as the reference (Ref) year. The top panel indicates the rate ratio for serious adverse events attributed to Definity™ as a function of total adverse events reported to FAERS. The bottom panel indicates the rate ratio for serious adverse events attributed to Definity™ as a function of overall serious adverse events reported to FAERS. *Confidence intervals are calculated using the 0.5 continuity correction to ensure finite intervals are reported.

Supplemental Table 12: Overall Adverse Event Reports to FAERS Attributed to Ultrasound Enhancing Agents by Year Compared with Gadolinium and Iodinated Contrast Agents and Neulasta™

Year	Total UEA reports (N = 4093)	Total Iodinated Contrast Reports (N = 10,115)	Total Gadolinium Contrast Reports (N = 6,286)	Total Neulasta™ (N = 72,255)	Total Reports to FAERS (N = 21,960,760)
2014	65	1134	583	2046	1198421
2015	224	1107	544	1169	1719763
2016	219	538	620	4440	1683307
2017	204	566	691	7204	1804751
2018	241	831	710	12501	2139736
2019	281	790	616	6399	2175069
2020	244	840	582	10295	2211092
2021	508	1029	594	9336	2344116
2022	703	1044	410	8102	2360165
2023	736	1028	412	6376	2212442
2024	668	1208	524	4387	2111898

Legend: Shown are the total number of adverse event reports reported to the U.S. Food and Drug Administration Adverse Event Reporting System (FAERS) along with the numbers attributed to ultrasound enhancing agents (UEAs), iodinated contrast, and gadolinium based contrast (both as negative controls) and pegfilgrastim (Neulasta™, positive control), from 2014-2024 by year. N = number of reports.

Supplemental Table 13: Rates of Total Adverse Reaction Rate Attributed to Ultrasound Enhancing Agents by Year Compared with Gadolinium and Iodinated Contrast Agents and Neulasta™

Year	Total UEA reports per 10,000	Total Iodinated Contrast Reports per 10,000	Total Gadolinium Contrast Reports per 10,000	Total Neulasta™ Reports per 10,000
2014	0.54	9.46	4.86	17.07
2015	1.30	6.44	3.16	6.80
2016	1.30	3.20	3.68	26.38
2017	1.13	3.14	3.83	39.92
2018	1.13	3.88	3.32	58.42
2019	1.29	3.63	2.83	29.42
2020	1.10	3.80	2.63	46.56
2021	2.17	4.39	2.53	39.83
2022	2.98	4.42	1.74	34.33
2023	3.33	4.65	1.86	28.82
2024	3.16	5.72	2.48	20.77
p-value for trend	*< 0.001*	*< 0.001*	*< 0.001*	*< 0.001*

Legend: Shown are rates of total adverse events attributed to ultrasound enhancing agents (UEAs) versus iodinated and gadolinium contrast (as negative controls) and pegfilgrastim (Neulasta™, positive control) and reported to U.S. Food and Drug Administration Adverse Event Reporting System (FAERS) from 2014-2024 by year. Rates are all provided per 10,000 reports. Additionally provided are p-values for Poisson trend tests across years.

Supplemental Table 14: Relative Risk of Total Adverse Events to UEAs, Iodinated Contrast, and Gadolinium Contrast, and Neulasta™ Reported to FAERS by Year

Year	Rate Ratio (95% CI) for UEAs	Rate Ratio (95% CI) for Iodinated Contrast	Rate Ratio (95% CI) for Gadolinium Contrast	Rate Ratio (95% CI) for Neulasta™
2014	Ref	Ref	Ref	Ref
2015	2.40 (1.82-3.17)	0.68 (0.63-0.74)	0.65 (0.58-0.73)	1.02 (1.02-1.02)
2016	2.40 (1.82-3.16)	0.34 (0.30-0.37)	0.76 (0.68-0.85)	1.04 (1.04-1.05)
2017	2.08 (1.58-2.76)	0.33 (0.30-0.37)	0.79 (0.70-0.88)	1.06 (1.06-1.07)
2018	2.08 (1.58-2.73)	0.41 (0.38-0.45)	0.68 (0.61-0.76)	1.09 (1.08-1.10)
2019	2.38 (1.82-3.12)	0.38 (0.35-0.42)	0.58 (0.52-0.65)	1.11 (1.10-1.12)
2020	2.03 (1.55-2.67)	0.40 (0.37-0.44)	0.54 (0.48-0.61)	1.13 (1.12-1.15)
2021	4.00 (3.09-5.17)	0.46 (0.43-0.50)	0.52 (0.46-0.58)	1.16 (1.14-1.18)
2022	5.49 (4.26-7.08)	0.47 (0.43-0.51)	0.36 (0.31-0.41)	1.18 (1.16-1.21)
2023	6.13 (4.76-7.90)	0.49 (0.45-0.53)	0.38 (0.34-0.43)	1.21 (1.18-1.23)
2024	5.83 (4.52-7.52)	0.60 (0.56-0.66)	0.51 (0.45-0.57)	1.23 (1.20-1.26)

Legend: Shown are the rate ratios and 95% confidence intervals (CIs) for the relative risk of total adverse events attributed to ultrasound enhancing agents (UEAs), iodinated and gadolinium contrast (as negative controls) and pegfilgrastim (Neulasta™, positive control) reported to the U.S. Food and Drug Administration Adverse Event Reporting System (FAERS) from 2014-2024 by year. Results are derived from Poisson regression using 2014 as the reference (Ref) year. All comparisons compared to the reference year are significant at a $p < 0.001$ value and thus p-values are not reported.

Supplemental Table 15: Number of Adverse Event Reports to UEAs at Piedmont, 2021-2024 by Severity of Reaction

Year	Total Reports (N = 99)	Serious Reports (N = 39)	Non-Serious Reports (N = 60)
2021	10	3	7
2022	18	9	9
2023	40	21	19
2024	31	6	25

Legend: Shown is a table displaying the numbers of serious and non-serious adverse events (AEs) across the Piedmont Health System in Georgia, 2021-2024, by year, overall mirroring national trends.
